# Mid-gestation serum lipidomic profile associations with spontaneous preterm birth are influenced by body mass index

**DOI:** 10.1371/journal.pone.0239115

**Published:** 2020-11-17

**Authors:** Kamil Borkowski, John W. Newman, Nima Aghaeepour, Jonathan A. Mayo, Ivana Blazenović, Oliver Fiehn, David K. Stevenson, Gary M. Shaw, Suzan L. Carmichael

**Affiliations:** 1 West Coast Metabolomic Center, Genome Center, University of California-Davis, Davis, CA, United States of America; 2 United States Department of Agriculture-Agriculture Research Service-Western Human Nutrition Research Center, Davis, CA, United States of America; 3 Department of Nutrition, University of California-Davis, Davis, CA, United States of America; 4 Department of Anesthesiology, Pain, and Perioperative Medicine, Stanford University School of Medicine, Stanford, CA, United States of America; 5 Department of Pediatrics, Stanford University School of Medicine, Stanford, CA, United States of America; 6 Department of Biomedical Data Sciences, Stanford University School of Medicine, Stanford, CA, United States of America; University of Pittsburgh, UNITED STATES

## Abstract

Spontaneous preterm birth (sPTB) is a major cause of infant morbidity and mortality. While metabolic changes leading to preterm birth are unknown, several factors including dyslipidemia and inflammation have been implicated and paradoxically both low (<18.5 kg/m^2^) and high (>30 kg/m^2^) body mass indices (BMIs) are risk factors for this condition. The objective of the study was to identify BMI-associated metabolic perturbations and potential mid-gestation serum biomarkers of preterm birth in a cohort of underweight, normal weight and obese women experiencing either sPTB or full-term deliveries (n = 102; n = 17/group). For this purpose, we combined untargeted metabolomics and lipidomics with targeted metabolic profiling of major regulators of inflammation and metabolism, including oxylipins, endocannabinoids, bile acids and ceramides. Women who were obese and had sPTB showed elevated oxidative stress and dyslipidemia characterized by elevated serum free fatty acids. Women who were underweight-associated sPTB also showed evidence of dyslipidemia characterized by elevated phospholipids, unsaturated triglycerides, sphingomyelins, cholesteryl esters and long-chain acylcarnitines. In normal weight women experiencing sPTB, the relative abundance of 14(15)-epoxyeicosatrienoic acid and 14,15-dihydroxyeicosatrienoic acids to other regioisomers were altered at mid-pregnancy. This phenomenon is not yet associated with any biological process, but may be linked to estrogen metabolism. These changes were differentially modulated across BMI groups. In conclusion, using metabolomics we observed distinct BMI-dependent metabolic manifestations among women who had sPTB. These observations suggest the potential to predict sPTB mid-gestation using a new set of metabolomic markers and BMI stratification. This study opens the door to further investigate the role of cytochrome P450/epoxide hydrolase metabolism in sPTB.

## Introduction

Preterm birth, i.e. delivery at <37wk gestation, is a major cause of infant morbidity and mortality, and accounts for 1 in 10 or ~400,000 births per year in the United states and ~11% of births worldwide or ~15 million births annually [1]. Part of the overall occurrence of preterm birth can be attributed to maternal or fetal conditions requiring medical intervention to facilitate delivery before term. However, risk factors and etiology of the largest population burden of preterm birth, spontaneous preterm birth (sPTB), remain largely unexplained [2]. As parturition is associated with an exquisitely regulated inflammatory process that promotes uterine contractility, cervical maturation and amniotic membrane rupture, pathophysiological priming of the inflammatory response by a variety of systemic stressors may increase the likelihood of sPTB [3, 4]. Vaginal infection, chronic inflammation, and inflammation-associated conditions like obesity and hypertension place a pregnancy at risk [5–7]. In fact early- to mid-gestation markers of low grade systemic inflammation, including increased pro-inflammatory cytokines interleukin (IL)-6, IL-8 and c-reactive protein and decreased anti-inflammatory IL-10, have been detected in women experiencing sPTB [8–11]. Notably, body mass indices (BMIs) for obesity (>30 kg/m^2^) and underweight (<18.5 kg/m^2^) relative to the normal range increase risks of sPTB [12–16], and the association of BMI with inflammation also exhibits a “U-shaped” behavior [17].

The inflammatory response is a multifaceted and coordinated system of interacting proteins and small molecule mediators with variability derived from both environmental and genetic influences [18]. Mechanistic associations between inflammation and sPTB have largely focused on protein level changes including cytokines [16], matrix metalloproteinases (MMPs) [19, 20], tissue inhibitors of MMPs [20], and tissue plasminogen activator [21]. Metabolomic investigation of circulating metabolites can provide a broader overview of changes in metabolism [22, 23] and inflammatory responses [24, 25]. Recent efforts have probed the metabolome for biomarkers of sPTB and clues to the etiology of the condition [26, 27]. While few of these studies have investigated maternal blood, those that have observe consistent differences in circulating lipids, with some reporting lipid mediator differences in plasma that can predict sPTB sub-type [26–28]. Among lipid mediators of inflammation, oxylipins, endocannabinoids, bile acids, and ceramides have each been implicated in the pathologies associated with preterm delivery [28–33]. Importantly, while the serum levels of these metabolites increase with BMI [34–36], this increase is associated with poor metabolic health (i.e. diabetes status) rather than obesity [37].

We designed the current study to specifically investigate BMI-associated metabolic perturbations and potential mid-gestational serum biomarkers for their associations with sPTB-risk in a cohort of underweight, normal weight, and obese women. To accomplish this goal, we have combined untargeted metabolomics and lipidomics with targeted metabolic profiling of non-esterified oxylipins, endocannabinoids, bile acids, ceramides and polyunsaturated fatty acids (PUFA) in mid-gestation serum samples.

## Materials and methods

### Subjects

Serum samples were collected from pregnant women in the gestational timing of 15–17 weeks by the California Biobank Program. This Biobank stores serum remaining after the California Prenatal Screening Program has conducted mid-gestation screening for fetal conditions such as trisomies and neural tube defects from women residing in selected CA counties: Orange, San Diego, Fresno, Madera, Merced, San Benito, Monterey, Kings, Tulare, Inyo, and Mono. Hospital discharge information from 2007 to 2011 were used to identify women with collected samples that were appropriate for the study. We identified nulliparous women who were linked to singleton livebirths birth certificate data provided by the Office of Statewide Health Planning and Development. Inclusion criteria were based on BMI [underweight (<18.5 kg/m2); normal (18.5–24.9 kg/m2); and obese (≥30 kg/m2)] and gestational age (sPTB <31 weeks; full-term birth (FTB) >37 weeks). BMI was based on self-reported weight and height and gestational age on best obstetric estimate, both as reported on the birth certificate. A review of self-reported maternal pre-pregnancy weight and weight change found that misclassification generally does not induce bias in associations with perinatal outcomes [38]. To reduce heterogeneity among all preterm births, we restricted the study to sPTB, i.e., those with preterm premature rupture of membranes, premature labor, or tocolytics based on information from the birth certificate or ICD-9 codes from the delivery hospitalization record. A list of births meeting these inclusion criteria was sent to the Biobank program to identify births with available serum samples and randomly select a sub-set from the following groups for our study: underweight sPTB (n = 288); underweight FTB (n = 11,430); normal weight sPTB (n = 366); normal weight FTB (n = 115,907); obese sPTB (n = 263); obese FTB (n = 28,510). From the available samples, we randomly selected 17 from each group for the current analysis. The sample group criteria and numbers selected were based in part on sample availability and budgetary constraints. Demographic information was derived from the birth certificate. Maternal race/ethnicity was categorized as non-Hispanic White, non-Hispanic Black, Asian, Pacific Islander, Hispanic, and missing. Maternal education was categorized as some high school or less, high school graduate or equivalent, some college, college degree or more, and missing. Maternal age (years) was included as a continuous variable. The study was approved by the California Committee for the Protection of Human Subjects, protocol number 15-01-1835. Data were analyzed anonymously.

### Serum targeted metabolomics

Quantitative targeted metabolomic profiling was performed using UPLC-MS/MS based methods in the presence of isotopically labeled or rare internal standards at the West Coast Metabolomics Center Lipid Mediators Research Laboratory (https://metabolomics.ucdavis.edu/research-labs/newman-laboratory-for-lipid-mediators). A complete list of extraction surrogates and their exact concentrations are presented in the **S1 Table**.

Oxylipins, endocannabinoids, PUFA and bile acids from 40 μL of serum were quantified after methanol/acetonitrile (1:1 v/v) protein precipitation [39]. Briefly, 40 μL of serum was mixed with 5 μL BHT/EDTA (1:1 methanol:water), 10 μL of 0.5 μM deuterated oxylipin, endocannabinoid and PUFA surrogates and 20 μL of 1 μM deuterated bile acid surrogates in methanol. Next, serum was homogenized by the vigorous addition of 200 μL of 1:1 methanol:acetonitrile containing 100 nM 1-cyclohexyl ureido, 3-dodecanoic acid (CUDA; Sigma, St. Louis MO) and 1-phenyl ureido 3-hexanoic acid (PUHA; kind gift from Dr. B.D. Hammock, University of California Davis). The homogenate was centrifuged at 15000 rcf for 10 min and the supernatant was filtered at 0.1 μm PVDF membranes (MiliporeSigma, Burlington, MA) at 1000 rcf for 5 min and collected for mass spectrometry analysis.

NS-ceramides were isolated and quantified using minor modifications of published procedures [40, 41]. Briefly, 100 μL of serum was mixed with 5 μL BHT/EDTA (1:1 methanol:water), 10 μL of 1 μM odd-chain length ceramide surrogates, and mixed with 410 μL of 2-propanol, followed by 520 μL of cyclohexane. Organic and aqueous phase splits were accomplished with the addition of 413 μL LC-MS grade water and 57 μL of 1 M ammonium acetate, and centrifugation at 10,000 rcf for 5 min. The upper organic phase was retrieved and dried under vacuum at 15 Hg for 10 min and residues were reconstituted in 100 μL of 1:1 methanol: acetonitrile containing 100 nM CUDA and PUHA.

Residues in extracts were separated on a 2.1 mm x 150 mm, 1.7 μm BEH C18 column (Waters, Milford, MA) for oxylipins and endocannabinoids analysis, 2.1 mm x 100 mm, 1.7 μm BEH C18 column (Waters) for bile acids analysis and 2.1 mm x 150 mm, 1.7 μm BEH C8 column (Waters) for ceramides analysis and detected by electrospray ionization with multi reaction monitoring on a API 6500 QTRAP (Sciex; Redwood City, CA) and quantified against 7–9 point calibration curves of authentic standards using modifications of previously reported methods [37, 42]. A complete list of metabolites together with groups means and p-values are provided in the **S2 Table**.

### Serum untargeted metabolomics

Semi-quantitative, untargeted metabolomic profiling was performed in the West Coast Metabolomics Center Central Services Core (https://metabolomics.ucdavis.edu/core-services/assays-and-services) as previously described to provide semiquantitative analysis of complex lipids and biogenic amines [43, 44]. Serum lipids were extracted from 40 μL of serum using biphasic solvent system of 300 μL of cold methanol containing odd chain and deuterated lipid internal standards: [LPE(17:1), LPC(17:0), PC(12:0/13:0), PE(17:0/17:0), PG(17:0/17:0), d7-cholesterol, SM(d18:1/17:0), Cer(d18:1/17:0), sphingosine(d17:1), DG(12:0/12:0/0:0), DG(18:1/2:0/0:0), and d5-TG-(17:0/17:1/17:0)], 1000 μL methyl tertbutyl ether (MTBE) containing CE(22:1) internal standard, and 250 μL of water. Lipid standards were purchased from Avanti Polar Lipids (Alabaster, AL USA). A 100 μL aliquot of the organic phase was dried under vacuum and subsequently reconstituted in 100 μL of methanol/toluene (9:1, v/v) containing CUDA (150 ng/mL) as an internal standard prior to LC-MS analysis. Biogenic amines were retrieved from the aqueous phase of the lipid extraction procedure. Samples were dried under the vacuum and rinsed with (1:1, v/v) acetonitrile: water to remove proteins and dried again under vacuum. Samples were reconstituted for Hydrophilic Interaction Liquid Chromatography (HILIC) mass spectrometry analysis in (80:20, v/v) acetonitrile:water solution containing CUDA (150 ng/mL) and deuterated internal standards. All measurements were carried out on a Thermo Q Exactive mass spectrometer. For complex lipids, 1 μL of extracts were separated on a Waters Acquity UPLC CSH C18 column (100 × 2.1 mm; 1.7 μm) coupled to an Acquity UPLC CSH C18 VanGuard precolumn (5 × 2.1 mm; 1.7 μm). The column was maintained at 65°C at a flow rate of 0.6 mL/min. The positive ionization mobile phases consisted of (A) acetonitrile:water (60:40, v/v) with ammonium formate (10 mM) and formic acid (0.1%) and (B) 2-propanol:acetonitrile (90:10, v/v) with ammonium formate (10 mM) and formic acid (0.1%). The negative ionization mobile phases consisted of (A) acetonitrile: water (60:40, v/v) with ammonium formate (10 Mm) and (B) 2-propanol: acetonitrile (90:10, v/v) with ammonium formate (10 mM). The separation was conducted under the following gradient: 0 min 15% B; 0−2 min 30% B; 2−2.5 min 48% B;2.5−11 min 82% B; 11−11.5 min 99% B; 11.5−12 min 99% B; 12−12.1 min 15% B; 12.1−15 min 15% B. For biogenic amines, hydrophilic interaction liquid chromatography (HILIC)-Q Exactive MS/MS data acquisition was performed. 1 μL of samples were separated on a Waters Acquity UPLC BEH Amide column (150 × 2.1 mm; 1.7 μm) coupled to an Acquity UPLC BEH Amide VanGuard precolumn (5 × 2.1 mm; 1.7 μm). The column was maintained at 45°C with a flow rate of 0.4 mL/min. The mobile phases consisted of (A) water with ammonium formate (10 mM) and formic acid (0.125%) and (B) acetonitrile: water (95:5, v/v) with ammonium formate (10 mM) and formic acid (0.125%). The separation was conducted under the following gradient: 0 min 100% B; 0−2 min 100% B; 2−7.7 min 70% B; 7.7−9.5 min 40% B; 9.5−10.25 min 30% B; 10.25−12.75 min 100% B; 12.75−17 min 100% B. The Q Exactive MS was operated using positive and negative mode electrospray ionization (ESI HILIC) with the following parameters: Mass range, 60–900 m/z; Sheath gas flow rate, 60; Aux gas flow rate, 25; Sweep gas flow rate, 2; Spray Voltage (kV) 3.6; Capillary temp, 300°C; S-lens RF level, 50; Aux gas heater temp, 370°C. Full MS parameters: Microscans—1; Resolution—60,000; AGC target - 1e6; Maximum IT - 100ms; Number of scans—1; Spectrum data type—Centroid. Data dependent MS2 parameters: Microscans—1; Resolution—15,000; AGC target - 1e5; Maximum IT—50 ms; Loop count—4; MSX count—1; TopN—4; Isolation Window—1.0 m/z; Isolation offset—0.0 m/z; (N)CE / stepped (N)CE—20, 30, 40; Spectrum data type—Centroid. The LC-MS/MS data were analyzed by MS-DIAL software [45]. Metabolite annotations were achieved using a combination of different tools. Metabolomics Standards Initiative (MSI) has defined levels of compound annotation which we applied in this paper [46]. On MSI level 1 we used a HILIC-MS/MS library of 1,400 authentic standards including retention time, precursor mass and MS/MS spectra. All spectra, retention times and chromatography conditions are freely available at MassBank of North America (http://massbank.us). Search windows were used as follows: 0.1 min RT tolerance (for the alignment of peaks), 0.01 Da tolerance for the precursor masses and 0.05 Da tolerance for the MS/MS spectral matching. Similarly, we used lipid retention times and MS/MS spectra for lipidomics identifications [47]. On MSI level 2, we annotated compounds that did not trigger MS/MS fragmentations in data dependent mode but that were still identified based on accurate mass and retention time using the HILIC-MS/MS library in addition to manually curated lipid retention times. For MSI level 3 annotations, we annotated compounds based on the MS1 information only using MS-DIAL software which has integrated Seven Golden Rules within [45, 48].

### Statistical analysis

All statistical tests were performed using JMP Pro 14.1 (JMP, SAS institute, Cary, NC). Prior to analysis, metabolite outliers were detected and removed using the robust Huber M test and missing data were imputed using multivariate normal imputation. Data imputation was performed to facilitate multivariate data analysis but did not significantly change results observed with univariate analysis (e.g. t-tests). The total of 1.14% of the data were imputed with a number of missing values in a single variable not exceeding 25%. Data were then transformed to obtain normal distributions using Johnson’s transformation. All analyses were stratified by BMI and metabolite data were adjusted for maternal age and race/ethnicity. Our primary objective was to evaluate BMI—specific metabolic perturbation related to sPTB in underweight, normal weight and obese BMI categories, and both sample collection and analyses were designed in a BMI-stratified fashion. Secondary analyses were performed to verify the appropriateness of the stratification approach. A factorial analysis was used to evaluate interaction between BMI categories and birth groups, as well as a general birth group effect. This model included BMI category, birth category, BMI x birth category interaction as the fixed effect and mother’s age as a covariate. Further, ANCOVA was used to evaluate metabolite mean differences between FTB and sPTB while evaluating the potential use of BMI as either a continuous or categorical covariate.

#### Targeted data analysis

Differences in metabolite mean concentrations were assessed using t-tests with Benjamini-Hochberg false discovery rate (FDR) correction with a q = 0.2 [49]. The fold difference used in the oxylipin/endocannabinoid network visualizations were calculated by division of the sPTB group mean by that of the FTB group. Group means were calculated using normally distributed data. Differences in cytochrome p450 metabolites were assessed using factorial analysis with preterm status, BMI category and the preterm x BMI interaction as fixed effects. Additionally, sPTB effect in the individual BMI categories was assessed using contrast post-test. Additional contrast post-test was applied to determine the difference between normal weight versus underweight and obese FTB groups.

#### Untargeted data analysis

Variables were clustered using SAS (Cary, NC) developed algorithm that uses principal components analysis for variable grouping and cluster components (the linear combination of all variables in each cluster) were calculated for each individual cluster to reduce the dimensionality and collinearity of the data and to gain additional information about associations among variables. Clustering resulted in combining 1194 variables into 179 clusters. Cluster components were further used in t-test analysis to describe the sPTB effect in each BMI category independently. P values from the t-tests were FDR corrected with the q = 0.2, as described above. **S3 Table** describes cluster members as well as their correlations within the cluster, least square means (standard error range) and t-test p-values for both clusters and individual metabolites.

#### Predictive modeling

Stepwise logistic regression was applied using automated process in JMP to determine the minimum strongest predictors of preterm delivery and was applied separately for each BMI category. Only quantitative data (i.e. targeted metabolomics) were used to build the models, which includes oxylipins, endocannabinoids, ceramides and bile acids to ensure the maximal translational potential of the model. Model variable addition was stopped at the minimum Bayesian Information Criterion (BIC). Models were assessed by evaluation of the area under the receiver-operator curve (ROC-AUC).

## Results

### Study cohort demographics

Among the women included in this analysis, most were Hispanic (50%) or non-Hispanic white (26%), many had greater than high school education (45%), and the mean age was 26 years. Demographics are summarized in **S4 Table**.

### Obese women experiencing sPTB show higher autoxidation and pro-inflammatory lipids

**Fig 1:**
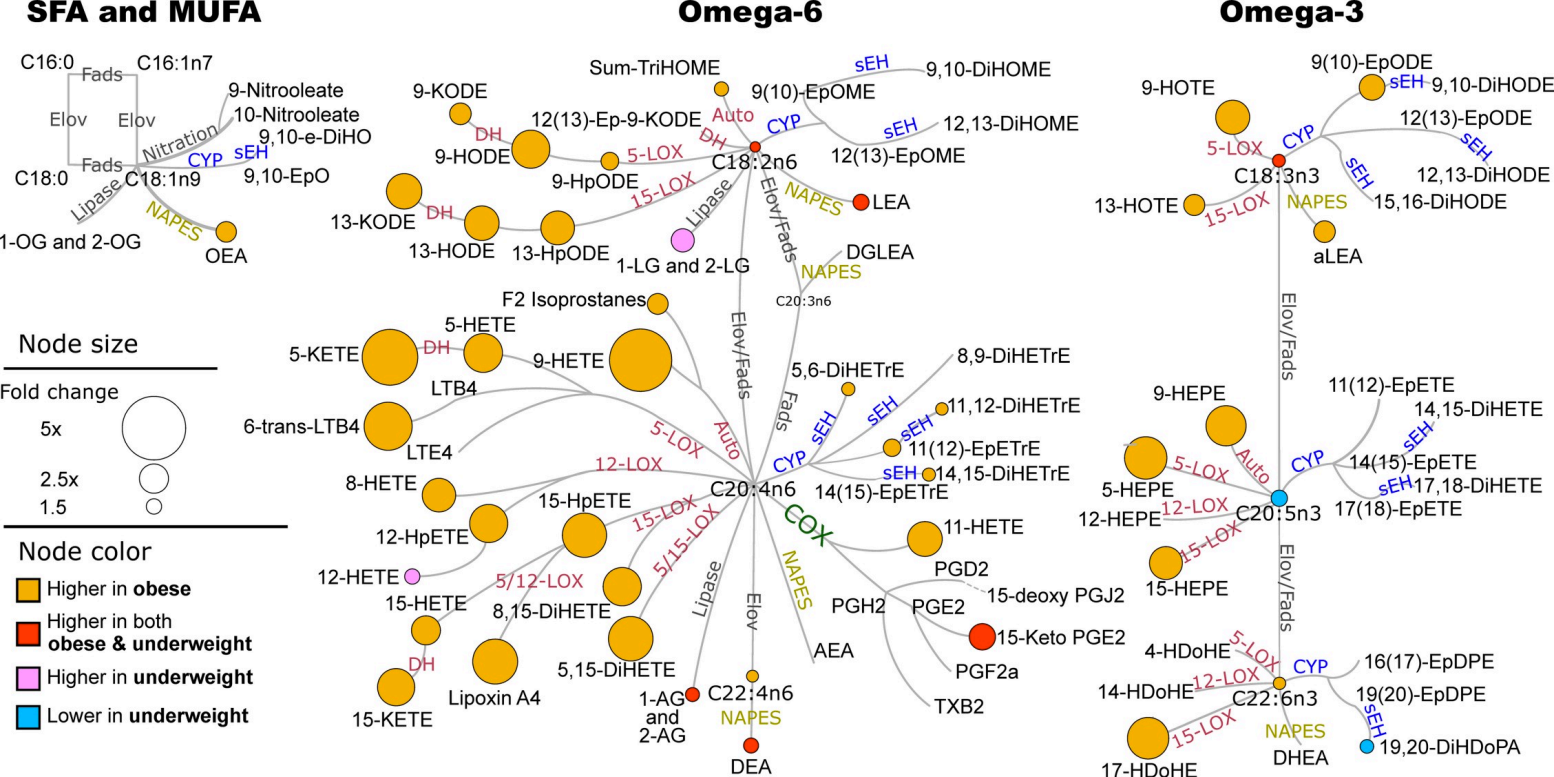
Differences in lipid mediators between sPTB and FTB in obese and underweight groups. Only differences with the t-test p<0.05 are shown. Network presents fatty acids metabolic pathway, including saturates and monounsaturates (SFA and MUFA) and omega 3 and omega 6 fatty acids with oxylipins and endocannabinoids synthesis pathway. Oxylipin metabolizing enzymes are colored by their class: red–lipoxygenase (LOX) and autoxidation pathway; blue–cytochrome p450 (CYP) epoxygenase; green–cyclooxygenase (COX); yellow–N-acylphosphatidylethanolamide-phospholipase D. Node size represents the fold change (sPTB/FTB) and the color represents the BMI group where the change was observed: orange–increased only in obese; red–increased in obese and underweight; pink–increased only in underweight; blue–decreased in underweight. Fads–fatty acid desaturase; Elov–fatty acids elongase; sEH–soluble epoxide hydrolase; DH–dehydrogenase. Saturated and monounsaturated fatty acids were not measured in this assay and are indicated only to visualize parent fatty acids for measured oxylipins and endocannabinoids. Means of individual metabolites and corresponding p-values are provided in S2 Table.

Targeted analysis detected 58 of 83 measured oxylipins, including products of cytochrome P450 (CYP), cyclooxygenase (COX), lipoxygenase (LOX), nitric oxide synthase metabolism and autoxidation, 10 of 21 endocannabinoids, and 5 of 5 PUFA (**Fig 1**). When testing for mean difference in targeted metabolites between sPTB vs FTB while using BMI as a continuous variable, only 7% had a p <0.05, but none passed an FDR correction at q = 0.2. Of note, similar results were obtained when adjusting for BMI as a categorical rather than continuous variable. On the other hand, when using BMI categories in factorial analysis, we found significant interaction between BMI and birth categories (11% with p <0.05) therefore, further analysis was stratified by BMI. T-test analysis was performed separately for normal weight, obese and underweight subjects. No significant differences in oxylipins, endocannabinoids or PUFAs were detected in the normal weight women, which are thus excluded from **Fig 1**. Among obese women, sPTB was associated with higher levels of LOX and autoxidation metabolites (orange nodes in **Fig 1**), regardless of the parent fatty acid’s carbon chain length and unsaturation. Although most hydroxy fatty acids (mainly LOX metabolites) are also products of autoxidation, arachidonic acid (C20:4n6—AA) derived 9-hydroxyeicosatetraenoic acid (9-HETE) and F2 isoprostanes, and eicosapentaenoic acid (C20:5n3—EPA) derived 9-hydroxyeicosapentaenoic acid (9-HEPE) are uniquely produced by autoxidation and were ~ 3 fold higher in obese sPTB subjects when compared to obese FTB subjects. Higher levels of six metabolites were associated with sPTB in both underweight and obese subjects: 18 carbon PUFA, linoleic and alpha linoleic acid (C18:2n6 and C18:3n3 –LA and aLA), LA and adrenic acid (C22:4n6) derived ethanolamides (LEA and DEA), AA derived monoacylglycerols (AGs) and AA derived prostaglandin (15-Keto PGE2). In addition, among underweight women, sPTB was associated with higher levels of AA derived 12-LOX metabolite (12-HETE), LA derived monoacylglycerols (LGs) and lower levels of EPA and docosahexaenoic acid (C22:6n3 –DHA) soluble epoxide hydrolase (sEH) metabolite 19,20 –dihydroxydocosapentaenoic acid (19,20-DiHDoPA). Targeted data group least square means and p-values are provided in the **S2 Table**.

### Normal weight women experiencing sPTB show shifts in arachidonic acid CYP/sEH metabolism

**Fig 2:**
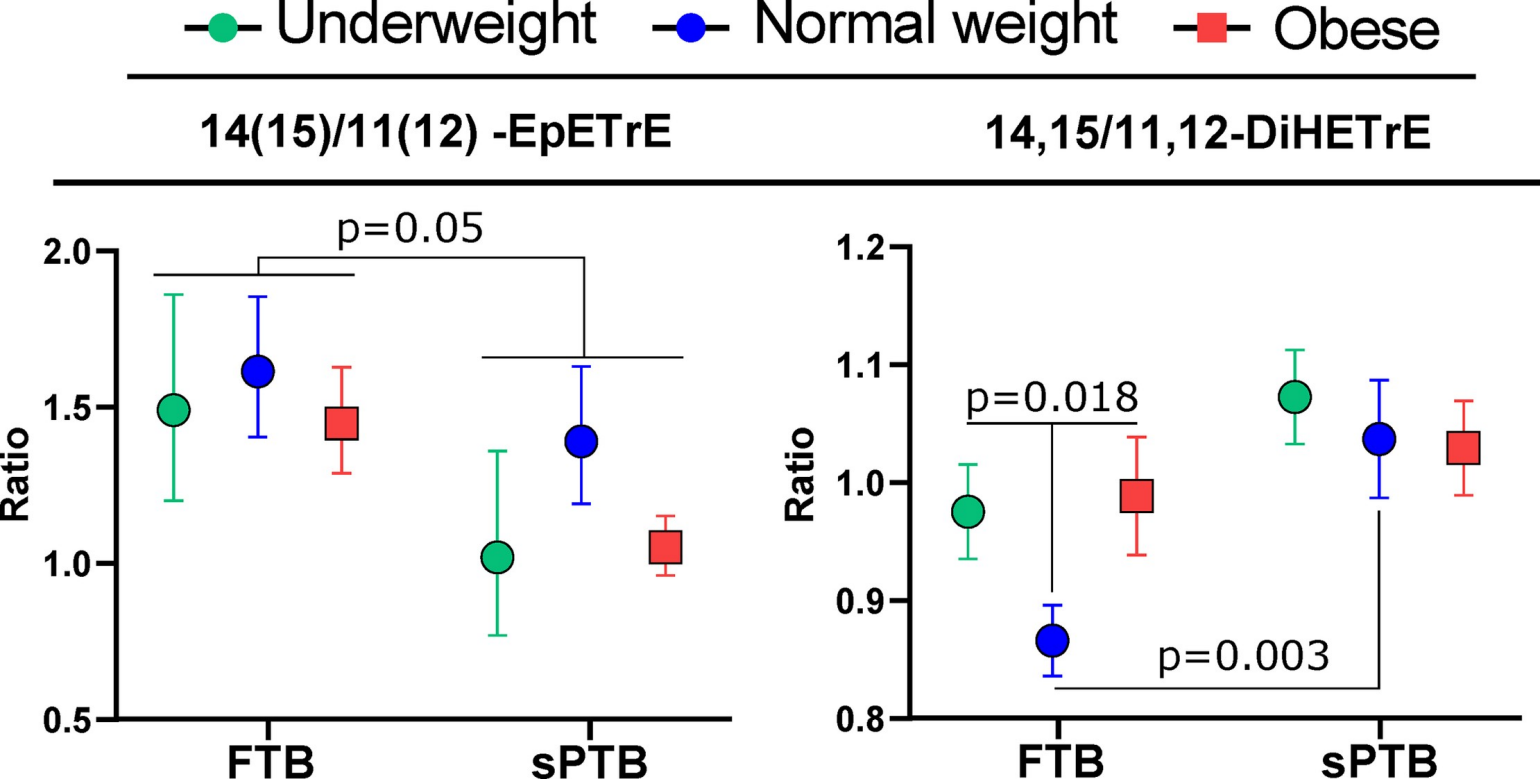
Regioisomer-specific differences in arachidonic acid cytochrome p450 (CYP) metabolism. Ratios of arachidonic acid CYP metabolite regioisomers (EpETrEs) and their soluble epoxide hydrolase (sEH)-dependent metabolites (DiHETrEs) stratified by BMI category and delivery group (sPTB and FTB). The regioisomeric ratios were calculated by dividing the concentrations of 14(15)-EpETrE or 14,15-DiHETrE by the concentration of 11(12)-EpETrE or 11,12-DiHETrE for epoxides and diols, respectively. The 14(15)/11(12) -EpETrE birth group effect was generated by a factorial analysis with BMI category, delivery group and the delivery x BMI interaction as fixed effects, and no significant interactions were detected. For the 14,15/11,12-DiHETrE ratio comparisons, the two p values reflect 1) the difference between FTB normal weight vs FTB obese and underweight, and 2) the difference between normal weight FTB vs normal weight sPTB. Error bars represent standard error. N = 17 per group. Point color: green–underweight; blue–normal weight; red–obese. EpETrE–epoxyeicosatrienoic acid; DiHETrE–dihydroxyeicosatrienoic acid.

Further, we investigated specific CYP- derived epoxy fatty acid and subsequent sEH-dependent dihydroxy fatty acid metabolites associated with sPTB (**Fig 2**). Relative to the FTB group, the sPTB group showed a lower ratio of 14(15)- to 11(12)-epoxyeicosatrienoic acid (EpETrE) in all three BMI categories. On the other hand, the corresponding ratio of 14,15- to 11,12-dihydroxyeicosatrienoic acid (DiHETrE) was elevated in the sPTB when compared to the FTB in normal weight, but not in obese or underweight women. Moreover, normal weight FTB subjects manifested a lower ratio of those diols than both obese and underweight FTB subjects (p = 0.018). The same behavior was observed for the ratio of 14,15- to 8,9-DiHETrE (**S1 Fig**). Independently, sPTB subjects had higher concentration of 11(12)-EpETrE and all AA-derived diols, when compared to FTB, in obese group only (**S1 Fig**).

### Both underweight and obese women who have sPTB show signs of dyslipidemia

**Fig 3:**
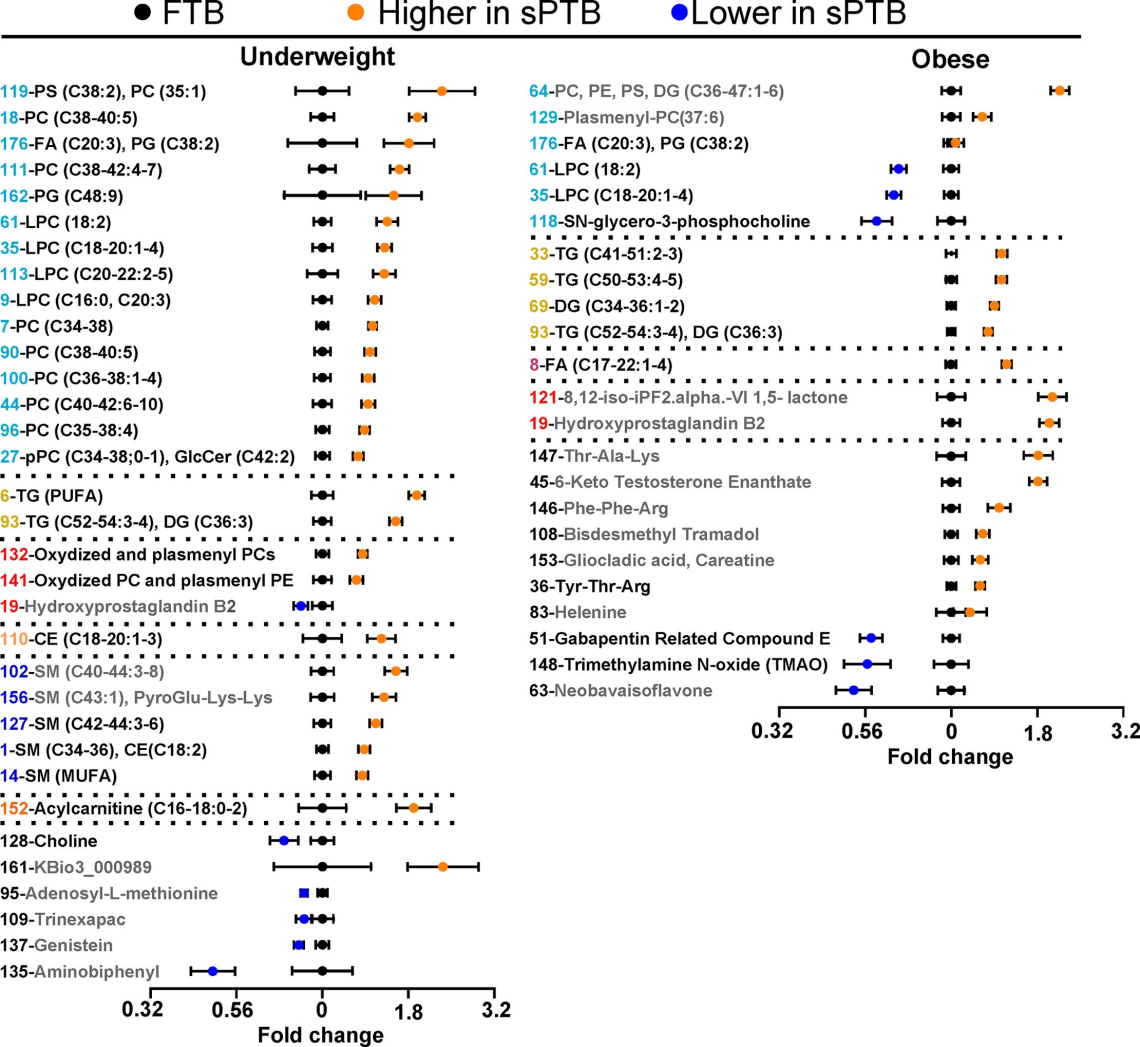
Differences in complex lipids and biogenic amines between sPTB and FTB deliveries. Results of the t-test analysis performed on variable cluster components, presented for women with underweight and obese BMI. Data are presented as the fold change from the FTB for each weight category. Each cluster is represented by a number and labeled with either a general description of variables within the cluster or the most representative metabolite. Clusters composed uniquely from metabolites identified only by parent mass (i.e. MSI level 3) are colored gray and should be interpreted with caution. A complete list of metabolites within each cluster with group means, p-values and MSI level are provided in the **S3 Table**. All displayed variables have p < 0.05 and passed FDR correction at q = 0.2. PC–phosphatidylcholine; PE–phosphatidylethanolamine; PS–phosphatidylserine; TG–triglyceride: DG–diglyceride; CE–cholesterol ester; SM–sphingomyelin; CA–cholic acid; FA–Fatty acids. Clusters are colored according their lipid classes: phospholipids and lysophospholipids–light blue; TG–yellow; oxidized lipids–red; SM–blue; CE–light orange; FA–purple; acylcarnitine–dark orange. Description of complex lipids—(total number of fatty acids carbon atoms: total number of double bounds). Error bars represent standard error. N = 17 per group.

To investigate broader metabolic perturbations in sPTB we used serum untargeted metabolomics of complex lipids and biogenic amines (**Fig 3**). We use variable clustering as a method of data reduction, to help summarize general differences in lipid and biogenic amines metabolism. **S3 Table** describes the 179 unique clusters of untargeted metabolomic features, including cluster membership, cluster correlations, means and p-values of cluster components and individual metabolites. Similar to the targeted data, when analyzing unstratified data using BMI as a continuous variable, we found only 6% of cluster components with p <0.05, but none passed an FDR correction at q = 0.2. On the other hand, when using BMI categories in factorial analysis, we found significant interaction between BMI and birth categories (10% with p<0.05) therefore, the further analysis was stratified by BMI. Similar to the oxylipin and endocannabinoid results, the normal weight sPTB group did not display significant differences and are not displayed in **Fig 3**. A summary of the numbers of identified lipids within each lipid class is presented in the **S5 Table**.

**Fig 4:**
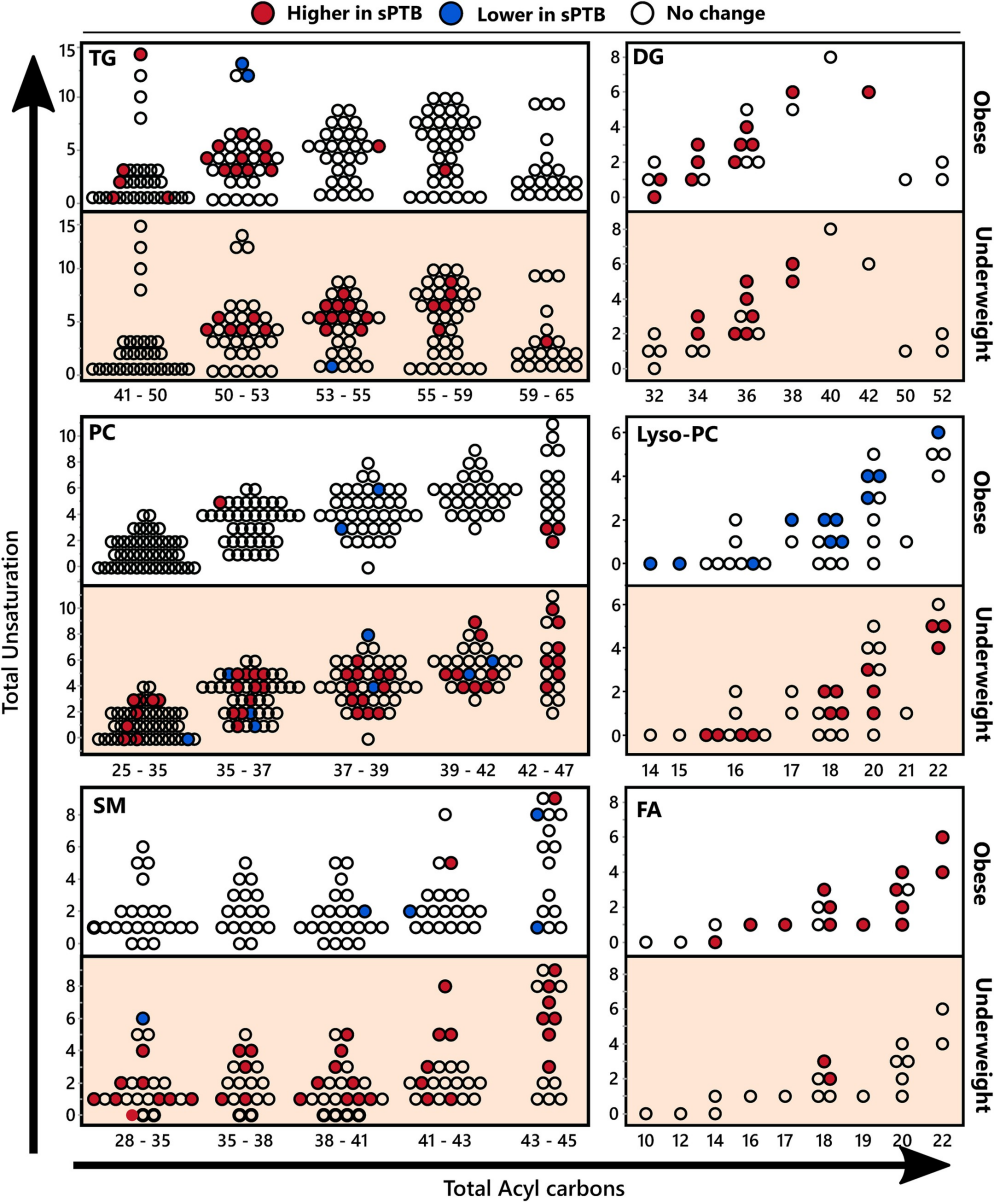
Serum complex lipids stratified by acyl chain length and number of double bonds. Differences in serum complex lipids between birth categories in obese and underweight women, stratified by acyl chain length and number of double bonds. Dots represent complex lipid species, and are positioned according to the total acyl chain unsaturation (y axis) and the total acyl chain length (x axis). Colors indicate significance and directionality of compared means (t-test p <0.05): red–higher in sPTB, blue–lower in sPTB, white–no change. TG–triglyceride; DG–diglyceride PS–phosphatidylserine; SM–sphingomyelin; FA–fatty acids. The number of detected individual complex lipids and the number of differences between sPTB and FTB for each chemical class are summarized in **S5 Table**. Means of individual lipids and corresponding p-values are provided in **S3 Table**.

With respect to complex lipids, differences observed between FTB and sPTB groups were unique for each BMI category, with only two commonly affected clusters, including cluster 93 (containing polyunsaturated triglycerides) and cluster 176 (containing homo-gamma-linolenic acid and phosphatidylglycerol). Both underweight and obese sPTB groups showed higher triglycerides and diglycerides (underweight group clusters—6, 93; obese group clusters—33, 59, 69, 93). As seen in **Fig 4**, which presents differences in individual lipids stratified by fatty acid moiety unsaturation and carbon chain length, this difference was manifested entirely in polyunsaturated species in the underweight group and showed a similar pattern in the obese group. The underweight sPTB group also showed higher levels of phospholipids and lysophospholipids (clusters 7, 9, 18, 27, 35, 44, 61, 90, 96, 100, 111, 113, 119, 162 and 176). In contrast, obese subjects experiencing sPTB had higher levels of only three phospholipid containing clusters (clusters 64, 129 and 176) and lower levels of lysophospholipids (clusters 35, 61 and 118). The differences in phospholipids did not manifest specificity towards fatty acid moiety unsaturation or chain length. Underweight subjects experiencing sPTB also displayed higher levels of sphingomyelins (clusters 1, 14, 102, 127 and 156), cholesterol esters (cluster 110) and acylcarnitines (cluster 152).

The sPTB association with oxidized lipids also differed between underweight and obese subjects. The obese sPTB group showed higher levels of F2 isoprostanes (consistent with the oxylipin analyses), while hydroxyprostaglandin B2 was lower in underweight subjects who experienced sPTB. The underweight sPTB group also showed higher levels of LA and aLA and lower levels of EPA, while obese subjects experiencing sPTB manifested higher levels of almost all fatty acids except for EPA, C14:1n5 and C14:0 and C12:0 (**Fig 4**). Differences in fatty acids levels observed in untargeted analysis are consistent with the targeted analyses (**Fig 1**).

With respect to biogenic amines, underweight sPTB group showed lower levels of choline, whereas obese sPTB subjects displayed lower levels of trimethylamine N-oxide (TMAO). Differences in sPTB were also observed among bile acids and ceramides analyzed using a targeted approach. THCSA was higher in the normal weight sPTB group (sPTB/FTB = 1.6, p = 0.004), while the underweight sPTB group had lower DCA levels (FTB/sPTB = 3, p = 0.033) and the obese sPTB group showed lower levels of GLCA (FTB/sPTB = 1.9, p = 0.023). Sphingosine (C18:1) was higher in both underweight (sPTB/FTB = 1.7, p = 0.005) and obese (sPTB/FTB = 1.8, p = 0.01) sPTB groups.

### sEH metabolite regioisomers are predictors of sPTB in normal and underweight women

Predictive models of sPTB were developed using stepwise logistic regression analyses of targeted metabolomic data (**Table 1**). Similar to the t-tests, the strongest models were obtained when stratified by BMI category (**RSq**_underweight_ = 0.60; **RSq**_normal weight_ = 1.0; **RSq**_obese_ = 0.67). Without BMI stratification a much weaker model was obtained (final model **RSq** = 0.24, with BMI itself not being selected by the stepwise logistic regression analysis). The elevated ratio of 14,15-/11,12-DiHETrE was selected as a factor in sPTB prediction for the underweight and normal weight, but not obese groups. Additionally, the sPTB model for normal weight subjects was characterized by a lower 12,13-/9,10-dihydroxy-octadeca(mono)enoic acid (DiHOME) ratio (i.e. the LA analogs of DiHETrEs) and a higher 14(15)-EpETrE/14,15-DiHETrE ratio. On the other hand, the sPTB in the obese group was best predicted by elevated levels of oleoylethnolamide (OEA), 5,6-DiHETrE and 6-trans-leukotriene B4 (6-trans-LTB4).

**Table 1:** Prediction of preterm delivery using serum lipid mediators.

BMI	Step	Parameter	p-value	sPTB/FTB	RSq	BIC	ROC AUC
**Underweight**	1	18:1 Sphingosine	0.0094	**+**	**0.143**	47.4	0.77
	2	14,15/11,12-DiHETrE	0.0332	**+**	**0.239**	46.4	0.79
	3	1/2_AG	0.0103	**+**	**0.379**	43.4	0.87
	4	Ceramide (d40:2)	0.0012	**-**	**0.601**	36.4	0.97
**Normal weight**	1	14,15/11,12-DiHETrE	0.0016	**+**	**0.218**	42.8	0.81
	2	12,13/9,10-DiHOME	0.0169	**-**	**0.343**	40.6	0.87
	3	THCSA	0.0017	**+**	**0.558**	34.2	0.94
	4	14(15)-EpETrE/DiHETrE	0	**+**	**1**	17.5	1.00
**Obese**	1	OEA	0	**+**	**0.367**	36.9	0.87
	2	5,6-DiHETrE	0.007	**+**	**0.522**	33.1	0.92
	3	6-trans LTB4	0.0094	**+**	**0.665**	29.9	0.97

Prediction of preterm delivery using serum levels of oxylipins, sphingolipids and ceramides, stratified by the BMI category. Stepwise logistic regression model (left side) shows the order of metabolites entered into the model. The “+” and “-” indicate the directionality of differences of means metabolite values observed between the birth categories. The model was stopped when the addition of next parameter would result in increase in corrected Bayesian Information Criterion (BIC). Receiver operating characteristic area under the curve (ROC AUC) is shown for each step of the model.

## Discussion

Preterm birth can lead to lifelong adverse health consequences and places significant strain on individuals, their families and the health care system. While our understanding of factors leading to this condition are still emerging [2], the early identification of subjects at elevated risk for sPTB offers opportunities to improve therapeutic strategies and enhance clinical outcomes. Body mass index either above (>30 kg/m^2^) or below (<18.5 kg/m^2^) the normal range increases the risk of sPTB [12–16], therefore the current study was designed to identify metabolic perturbations present in serum at mid-gestation that are associated with sPTB in women of low and high BMI. Findings highlight sPTB risk as a complex metabolic phenomenon that is influenced by BMI.

Inflammation is an important component of sPTB [16, 19–21], and many mediators of inflammation are known including oxylipins, endocannabinoids and bile acids. Oxylipins constitute a metabolite superclass formed by coordinated metabolic cascades with important inflammatory and immunomodulatory functions [50]. COXs lead to prostanoid and thromboid synthesis, LOXs produce hydroperoxides leading to an array of downstream products (e.g. mid-chain alcohols, ketones, leukotrienes, lipoxins, resolvins), and CYPs form epoxy and omega-hydroxy fatty acids, while sources of reactive oxygen species (e.g. NADPH oxidases, mitochondria, peroxisomes, environmental pollutants) lead to PUFA autooxidation producing racemic peroxide-associated products including alcohols and prostaglandins [51–53]. In general, prostanoids have both pro- and anti-inflammatory roles [54], epoxy fatty acids are anti-inflammatory, suppressing pro-inflammatory cytokine production [55], while mid-chain alcohols are pro-inflammatory and can induce pro-inflammatory cytokines [63, 64]. The acylethanolamides, which include a major class of endogenous cannabinoid receptor and peroxisome proliferator-activated receptor alpha (PPARα) ligands, also modulate inflammation, and have circulating concentrations that are modulated by acute inflammatory responses [56]. Notably, both underweight and obese women who experienced sPTB showed distinct oxylipin and endocannabinoid levels at mid-gestation, while women in the normal weight women manifested fewer sPTB-associated metabolomic excursions.

While obesity can directly influence the circulating metabolome, if these changes are themselves important factors in sPTB risk, an exacerbation of these effects would be expected in the obese sPTB group. In fact, elevated fatty acid ethanolamides and reduced lysophosphatidylcholines with unchanged levels of phosphatidylcholines are the features observed in obesity [63, 64], the same changes were found by us to be exacerbated in the obese sPTB group. Moreover, the obese sPTB group showed higher levels of PUFA mono-alcohols than the obese FTB group, including the autoxidation products 9-HETE and F2-isoprostanes. All detected mono-alcohols, non-vicinal diols (e.g. leukotrienes) and triols (e.g. lipoxins) correlated strongly with the autooxidative markers (**S6 Table**) supporting an autooxidative origin [57, 58]. A recent study of effects of long-term storage on the plasma metabolome reports general stability for up to 7yrs, with significant changes observed with additional time up to 16yrs [59]. Moreover, both preanalytical handling of the samples, and long- term storage prior to analysis can increase fatty acids alcohols and isoprostanes [60, 61], and it is possible that observed changes in autoxidation markers are not biological. With respect to oxylipins, stability for up to 15 months has been reported [62], but longer duration influences have not been reported. However, exploration of archived plasma has proven useful for oxylipin discovery efforts identifying metabolites with correlations to know inflammatory markers [63]. Therefore, we cannot explicitly confirm that lipid markers identified here were not artifactually generated, however, as the samples were selected at random with no difference in collection time between groups, we expect this to contribute to random error but not biased the results. Moreover, our findings are consistent with a recently published analysis of eicosanoids in subjects experiencing sPTB [27] also using samples stored for a prolonged period of time prior to analysis. Those findings indicate that obese individuals either experience oxidative stress, or their plasma lipids are more susceptible to oxidation, at mid-gestation, consistent with previous reports regarding obesity-associated oxidative stress [64], and the elevated production of PUFA mono-alcohols in obesity-associated disorders [37, 65]. Notably, both targeted and untargeted metabolomics showed higher levels of non-esterified fatty acids in the obese sPTB group, further supporting dyslipidemia and adipose tissue insulin resistance in these individuals [66]. Together, these findings are consistent with poorer metabolic health characterized by exacerbated mid-gestation dyslipidemia in obese women who experience sPTB relative to those obese women who deliver at term.

Underweight women who experienced sPTB also manifested signs of dyslipidemia but not oxidative stress. The underweight sPTB group had higher non-esterified 18 carbon PUFAs and lower EPA, along with higher levels of most complex lipid species. In particular, the majority of detected phospholipids were elevated, a change associated with insulin resistance in young adults [17]. While lipoprotein speciation was not directly measured, the elevated levels of triglycerides with strong correlations to phosphatidylcholine levels are consistent with increased very low density lipoprotein levels [67]. Beyond these findings, the underweight sPTB subjects also showed higher levels of long-chain acylcarnitines including AC18:1, AC18:2 and AC16:0 which have been reported to be increased in obese and diabetic subjects [68]. Moreover, as serum AC16:0 and AC18:1 are reported to be positively correlated with TNFα and IL-6 and associated with fatty liver disease [69], these changes may indicate inflammation and poor metabolic health in the underweight women. Changes observed in bile acids metabolism were also consistent with body weight associated changes [70]. Therefore, underweight women who experience sPTB also show signs of dyslipidemia that are distinct from the sPTB characteristics of the obese subjects.

While differences were seen between the obese and underweight sPTB groups, similarities were also noted. For instance, women who were underweight or obese and experienced sPTB both displayed specific enrichment in triglyceride species carrying polyunsaturated fatty acids. Dyslipidemia was previously described as a sPTB risk factor [71, 72]. Moreover, 15-keto-PGE2 was one of the few oxylipins with higher levels in both obese and underweight sPTB groups. This oxylipins is derived from PGE2 [73] and catabolized by prostaglandin reductase 2 [74]. 15-Keto PGE2 is an important PPARγ agonist during adipocyte differentiation [74]. PPARγ also regulates intracellular lipolysis in adipose tissue [75], and activated adipose PPARγ could explain the elevated free fatty acids in sPTB subjects. On the other hand, 15-Keto PGE2 was positively associated with better LPS-induced sepsis survival and the suppression of pro-inflammatory cytokines production in murine macrophages [76]. Since sPTB was associated with elevated levels of circulating pro-inflammatory cytokines, the increased 15-Keto PGE2 is consistent with a modulating anti-inflammatory response at mid-gestation. An immunosuppressive role of prostaglandins in inflammation resolution was previously reported [54]. Therefore, activation of the immune system including both the pro- and anti-inflammatory tones, are associated with sPTB risk in a weight independent fashion.

While few metabolites discriminated sPTB from FTB in normal weight individuals, those that did suggested subtle changes in mid-gestation CYP/sEH epoxy fatty acid metabolism resulting in shifted regioisomeric metabolite profiles. Notably, these isomeric shifts were consistent in both underweight and obese FTB women, when compared to the normal weight women, supporting a common shift in CYP/sEH epoxy fatty acid metabolism at the both extremes of BMI, groups that are at higher risk for sPTB. Epoxy fatty acids are generated by multiple CYP enzymes and are further converted to their corresponding vicinal diols by epoxide hydrolases [77, 78]. While CYP and epoxide hydrolases show some regioselective product formation and substrate selective respectively, isomeric changes in CYP metabolite profiles in circulation are poorly characterized and not yet associated with specific metabolic outcomes. However here, in the normal weight sPTB group we found a preferential production of the arachidonic acid-derived 14,15-DiHETrE over the 11,12- and 8,9-regioisomers, accompanied by a decrease in the ratio of 14(15)-/11(12)-EpETrE, precursors of the corresponding diols. Relative changes in individual EpETrEs were not significant. In fact, the 14,15-/11,12-DiHETrE ratio was a strong predictor of sPTB in normal weight and to a lesser extent in underweight subjects. These findings are consistent with the reported elevation of 11(12)-EpETrE, but not 14(15)-EpETrE in sPTB [27]. Additionally, the fact that normal weight subjects experiencing FTB had lower ratios of 14,15-/11,12-DiHETrE and 14,15-/8,9-DiHETrE than both the underweight and obese FTB groups suggests that body mass either modulates or masks the underlying biochemistry. Interestingly, the ratio of 14,15-/11,12-DiHETrE in ovarian follicular fluid was previously shown to be tightly correlated with plasma estradiol levels across the estrus cycle in the pig [79], and elevated plasma and amniotic estradiol is associated with sPTB in women [80], fostering a speculated connection between CYP metabolism, steroid hormone production and sPTB. Along these lines, several reports also suggest the involvement of epoxy fatty acids in the regulation of matrix metalloproteinases (MMPs) [81, 82]. MMPs are crucial for membrane rupture during delivery [83] and dysregulation of their expression was reported in sPTB [84]. Therefore, the potential connection between dysregulation of CYP metabolism, MMP and sPTB are intriguing in light of our observation of isomeric shifts in arachidonic acid-derived CYP metabolites. Understanding the sources of the observed alteration in DiHETrE regioisomeric abundance may provide novel approaches to reduce the risk of sPTB in the future.

This was a small study conducted using biobanked aliquots of collected serum samples collected at one timepoint in pregnancy, which have inherent limitations. As such, factors including fasting state and pre-analytical sample handling were not explicitly controlled. However, in our previous study simulating preanalytical sample handling of serum from the biobank used here, with the exception of lysophospholipids, all compounds described here as associated with sPTB were not affected [60]. Additionally, we have no reason to expect that sample handling would have differed between those women who experienced sPTB versus FTB, given that samples were collected prospectively, before the window of delivery of a liveborn infant. Additionally, due to the limitation in available samples, the range of gestational age in underweight sPTB group was broader than in obese and normal weight sPTB groups. The small group size precluded the robust validation of the reported predictive models, and our findings require validation in independent cohorts. Thus, the results should be applied elsewhere cautiously until such validation has been completed.

In summary, our results show distinct metabolic manifestations of the sPTB phenotype depending on the BMI category and provides further evidence for inflammatory and autooxidative origin of this phenomenon. More importantly, this work demonstrated the potential to predict sPTB at midterm using new set of metabolomic markers and stratification by BMI. This would be especially important for the mothers without weight related risk factors. Moreover, our findings point towards the potential role of a regioisomeric shift in CYP/epoxide hydrolase–dependent AA metabolism in sPTB risk. The regioisomeric shifts in this pathway have been rarely reported and are poorly understood and to our knowledge, not yet associated with any biological phenomenon. Finally, this study opens the door to further research on the role of CYP/epoxide hydrolase metabolism in sPTB which may involve steroid hormone and MMP metabolism.

## Supporting information

S1 Fig Differences in serum cytochrome P450 (CYP) metabolism between preterm birth (PTB) and full-term birth (FTB) women, stratified by the BMI categories. (TIF)

S1 Table Concentration of surrogates in surrogate spike solution. (XLSX)

S2 Table Targeted data quantification table. (XLSX)

S3 Table Untargeted data quantification table. (XLSX)

S4 Table Demographic representation of experimental groups. (DOCX)

S5 Table Number of detected individual complex lipids and the number of differences between sPTB and FTB for each chemical class. (DOCX)

S6 Table Spearman’s ρ correlations between non-vicinal diol (5,15-DiHETE), mono-alcohols derived from 15 and 12 LOX (15-HETE and 12-HETE respectively), leukotriene B4 (LTB4), lipoxin A4 (Variable column) and autoxidative markers, F2 isoprostanes and 9-HETE (By variable column). (DOCX)

## Abbreviations

BMI: body mass index
COX: Cyclooxygenase
CYP: Cytochrome P450
DiHETrE: Dihydroxyeicosatrienoic acid
DiHOME: Dihydroxy-octadeca(mono)eneoic acid
EpETrE: Epoxyeicosatrienoic acid
FTB: Full-term birth
HETE: Hydroxyeicosatetraenoic acid
IL: Interleukin
LOX: Lipoxygenase
MMPs: Matrix metalloproteinases
PUFA: Polyunsaturated fatty acids
she: Soluble epoxide hydrolase
sPTB: Spontaneous preterm birth
