# Supplementary figures and images for: Mid-gestation serum lipidomic profile associations with spontaneous preterm birth are influenced by body mass index
Source: PLoS One. 2020 Nov 17;15(11):e0239115. doi: 10.1371/journal.pone.0239115 (PMC7671555; doi:10.1371/journal.pone.0239115)

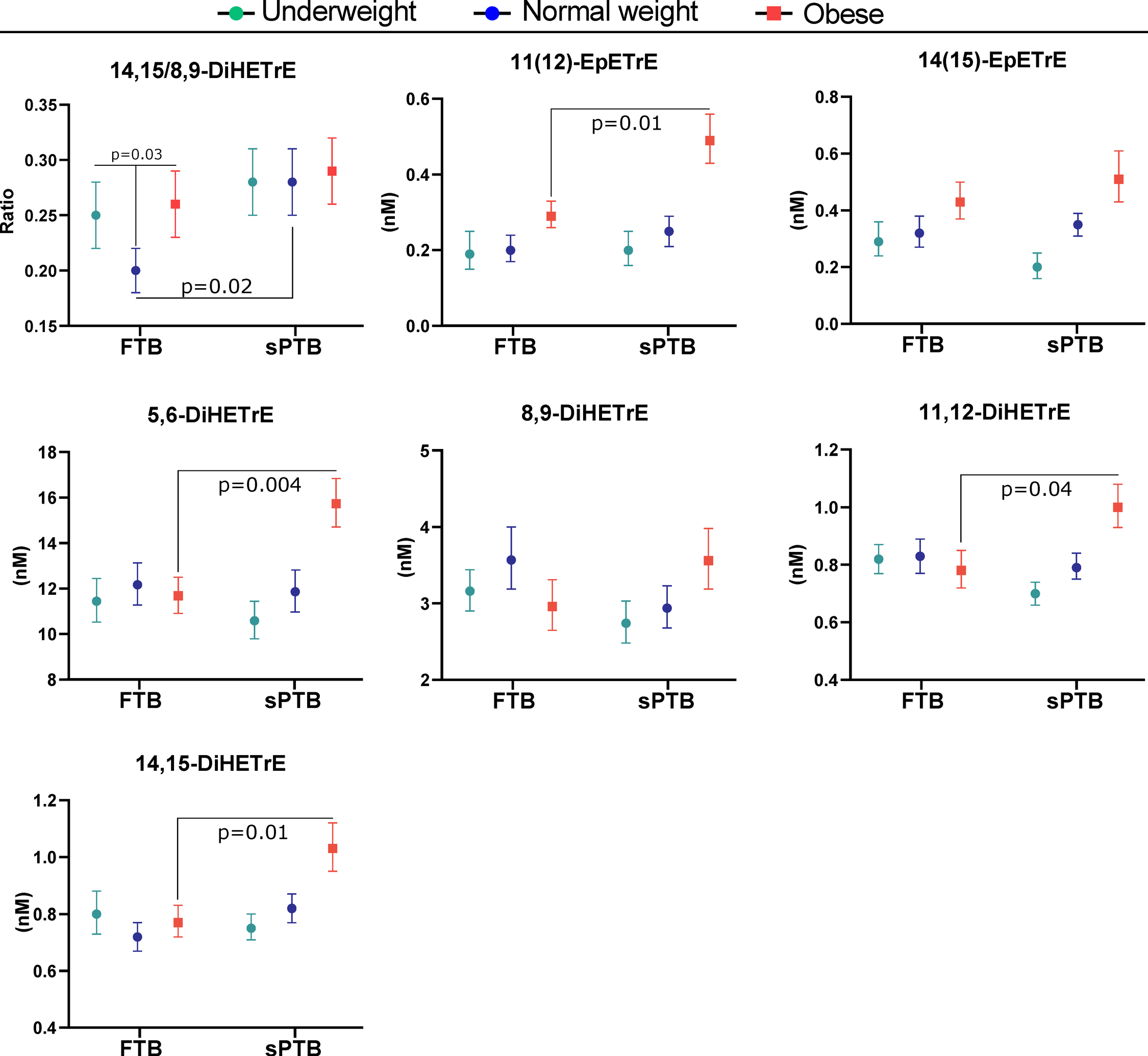

Supplement: S1 Fig — (TIF) [file pone.0239115.s001.tif]
