# Supplementary material for: Mid-gestation serum lipidomic profile associations with spontaneous preterm birth are influenced by body mass index
Source: PLoS One. 2020 Nov 17;15(11):e0239115. doi: 10.1371/journal.pone.0239115 (PMC7671555; doi:10.1371/journal.pone.0239115)
Supplement: S4 Table — (DOCX) [file pone.0239115.s005.docx]

**Table S4**. Demographic representation of experimental groups.*

|  | **All subjects** | **FTB** | | | **PTB** | | |
| --- | --- | --- | --- | --- | --- | --- | --- |
|  |  | **Underweight** | **Normal weight** | **Obese** | **Underweight** | **Normal weight** | **Obese** |
| **Total, *n*** | 102 | 17 | 17 | 17 | 17 | 17 | 17 |
| **Maternal race/ethnicity, *n (%)*** | | | | | | | |
| Non-Hispanic White | 26% | 35% | 29% | 35% | nc | nc | nc |
| Non-Hispanic Black | 5% | nc | nc | nc | nc | nc | nc |
| Asian or Pacific Islander | 19% | 47% | nc | nc | 29% | nc | nc |
| Hispanic | 50% | nc | 47% | 59% | 35% | 65% | 71% |
| **Maternal education, *n (%)*** | | | | | | | |
| High school diploma or less | 55% | 53% | <30% | 47% | >70% | 65% | 69% |
| Some college | 45% | 47% | >70% | 53% | <30% | 35% | 31% |
| **Maternal biometrics** | | | | | | | |
| Maternal Age, *mean* ± *std* | 25.56 ± 6.25 | 27.06 ± 6.55 | 26.53 ± 5.95 | 25.06 ± 5.46 | 23.59 ± 6.91 | 22.94 ± 5.62 | 28.18 ± 6.06 |
| Prepregnancy weight, *mean* ± *std* | 138.19 ± 43.04 | 100.71 ± 5.21 | 124.71 ± 14.4 | 179.76 ± 30.91 | 98.29 ± 9.94 | 126.18 ± 17.8 | 199.47 ± 27.02 |
| Height, *mean* ± *std* | 62.99 ± 2.79 | 64.18 ± 2.74 | 63.18 ± 2.58 | 61.65 ± 2.52 | 62.94 ± 3.05 | 63.59 ± 2.67 | 62.41 ± 2.83 |
| Prepregnancy BMI, *mean* ± *std* | 24.6 ± 7.88 | 17.23 ± 1.07 | 21.93 ± 1.77 | 33.15 ± 4.3 | 17.42 ± 0.81 | 21.9 ± 2.35 | 36 ± 4.43 |
| Gestational age (weeks), *mean* ± *std* |  | 39.24 ± 0.81 | 39.18 ± 0.91 | 39.23 ± 1.15 | 26.82 ± 3.07 | 24.71 ± 1.72 | 24.47 ± 1.97 |

* Percentages were calculated based on non-missing data; data were missing for <5% of women for race/ethnicity and education and there were no missing values for the other variables. For race/ethnicity, nc = not calculated due to cell size <5. For education, within study groups, approximate percentages are reported for some groups to avoid revealing cell sizes <5 (as noted by ‘<’ or ‘>’).
