# Supplementary material for: Mid-gestation serum lipidomic profile associations with spontaneous preterm birth are influenced by body mass index
Source: PLoS One. 2020 Nov 17;15(11):e0239115. doi: 10.1371/journal.pone.0239115 (PMC7671555; doi:10.1371/journal.pone.0239115)
Supplement: S5 Table — (DOCX) [file pone.0239115.s006.docx]

**S5 Table.** Number of detected individual complex lipids and the number of differences between PTB and FTB for each chemical class.

| **General lipid class** | **Lipid sub-class** | **Total detected** | **Differences PTB vs FTB** | |
| --- | --- | --- | --- | --- |
|  |  |  | **Underweight** | **Obese** |
| Acylcarnitine | | 6 | 3 | 0 |
| CE | | 14 | 5 | 1 |
| Ceramides | Ceramide | 13 | 1 | 2 |
|  | GlcCer | 6 | 3 | 0 |
| Phospholipids | PC | 166 | 61 | 8 |
|  | PE | 43 | 13 | 2 |
|  | PS | 9 | 3 | 2 |
|  | PG | 7 | 3 | 0 |
|  | Lyso-PC | 33 | 14 | 12 |
|  | Lyso-PE | 8 | 1 | 0 |
|  | Plasmenyl-PC | 29 | 7 | 2 |
|  | Plasmenyl-PE | 14 | 1 | 1 |
| Sphingolipids | SM | 96 | 38 | 6 |
|  | Sphingosine | 1 | 1 | 1 |
| Glicerides | TG | 151 | 23 | 19 |
|  | DG | 22 | 9 | 11 |
| Fatty acids | | 24 | 4 | 17 |
| **Total** | | **642** | **190** | **84** |
