# Supplementary material for: Mid-gestation serum lipidomic profile associations with spontaneous preterm birth are influenced by body mass index
Source: PLoS One. 2020 Nov 17;15(11):e0239115. doi: 10.1371/journal.pone.0239115 (PMC7671555; doi:10.1371/journal.pone.0239115)
Supplement: S6 Table — (DOCX) [file pone.0239115.s007.docx]

**S6 Table.** Spearman’s ρ correlations between non-vicinal diol (5,15-DiHETE), mono-alcohols derived from 15 and 12 LOX (15-HETE and 12-HETE respectively), leukotriene B4 (LTB4), lipoxin A4 (Variable column) and autoxidative markers, F2 isoprostanes and 9-HETE (By variable column).

| Variable | By variable | Spearman ρ | P value |
| --- | --- | --- | --- |
| 5_15_DiHETE | 9_HETE | 0.8895 | <.0001 |
| 12_HETE | 9_HETE | 0.3251 | 0.0009 |
| 15_HETE | 9_HETE | 0.8838 | <.0001 |
| Lipoxin A4 | 9_HETE | 0.954 | <.0001 |
| LTB4 | 9_HETE | 0.5785 | <.0001 |
| 5_15_DiHETE | F2 Isoprostanes | 0.7821 | <.0001 |
| 12_HETE | F2 Isoprostanes | 0.3034 | 0.002 |
| 15_HETE | F2 Isoprostanes | 0.8154 | <.0001 |
| Lipoxin A4 | F2 Isoprostanes | 0.8779 | <.0001 |
| LTB4 | F2 Isoprostanes | 0.5139 | <.0001 |
